# Loss of Caveolin-1 Accelerates Neurodegeneration and Aging

**DOI:** 10.1371/journal.pone.0015697

**Published:** 2010-12-23

**Authors:** Brian P. Head, Jason N. Peart, Mathivadhani Panneerselvam, Takaakira Yokoyama, Matthew L. Pearn, Ingrid R. Niesman, Jacqueline A. Bonds, Jan M. Schilling, Atsushi Miyanohara, John Headrick, Sameh S. Ali, David M. Roth, Piyush M. Patel, Hemal H. Patel

**Affiliations:** 1 Department of Anesthesiology, University of California San Diego, La Jolla, California, United States of America; 2 VA San Diego Healthcare System, San Diego, California, United States of America; 3 Department of Medicine, University of California, La Jolla, California, United States of America; 4 Heart Foundation Research Centre, Griffith University, Gold Coast, Queensland, Australia; 5 Gene Therapy Program, University of California San Diego, La Jolla, California, United States of America; Federal University of Rio de Janeiro, Brazil

## Abstract

**Background:**

The aged brain exhibits a loss in gray matter and a decrease in spines and synaptic densities that may represent a sequela for neurodegenerative diseases such as Alzheimer's. Membrane/lipid rafts (MLR), discrete regions of the plasmalemma enriched in cholesterol, glycosphingolipids, and sphingomyelin, are essential for the development and stabilization of synapses. Caveolin-1 (Cav-1), a cholesterol binding protein organizes synaptic signaling components within MLR. It is unknown whether loss of synapses is dependent on an age-related loss of Cav-1 expression and whether this has implications for neurodegenerative diseases such as Alzheimer's disease.

**Methodology/Principal Findings:**

We analyzed brains from young (Yg, 3-6 months), middle age (Md, 12 months), aged (Ag, >18 months), and young Cav-1 KO mice and show that localization of PSD-95, NR2A, NR2B, TrkBR, AMPAR, and Cav-1 to MLR is decreased in aged hippocampi. Young Cav-1 KO mice showed signs of premature neuronal aging and degeneration. Hippocampi synaptosomes from Cav-1 KO mice showed reduced PSD-95, NR2A, NR2B, and Cav-1, an inability to be protected against cerebral ischemia-reperfusion injury compared to young WT mice, increased Aβ, P-Tau, and astrogliosis, decreased cerebrovascular volume compared to young WT mice. As with aged hippocampi, Cav-1 KO brains showed significantly reduced synapses. Neuron-targeted re-expression of Cav-1 in Cav-1 KO neurons *in vitro* decreased Aβ expression.

**Conclusions:**

Therefore, Cav-1 represents a novel control point for healthy neuronal aging and loss of Cav-1 represents a non-mutational model for Alzheimer's disease.

## Introduction

Cognitive decline is emerging as one of the greatest health problems in the elderly population [1], [2]. Age alone increases the risk of stroke, Alzheimer's disease (AD), and other forms of dementia [2]. The risk of AD increases 14-fold between the ages of 65–85, and affects almost 47% over the age of 85 [3].

Multiple signaling pathways regulate neuronal survival and growth to facilitate the formation of synapses and this signaling is altered with age [4], [5], [6], [7]. Synapses are essential for learning, memory and the development of neurons in the CNS [8]. Receptors and associated proteins aggregate to mold and shape post-synaptic densities in order to permit high fidelity signal transduction leading to rapid regulation of neuronal function [9], [10], [11]. Understanding the basic pathophysiological mechanisms of cognitive decline and how the subcellular organization of signaling molecules is altered with cognitive decline could potentially yield novel therapeutic targets for neuronal aging and neurodegeneration.

Cholesterol is a major lipid component of synapses and a limiting factor in synapse development, synaptic activity, and neurotransmitter release [12]. Age-related impairments in the biosynthesis, transport, or uptake of cholesterol by neurons in the CNS may adversely affect development, plasticity, and synaptic circuitry associated with neurodegenerative diseases [13], [14], [15], [16], [17]. Membrane lipid rafts (MLR), discrete regions of the plasma membrane enriched in cholesterol, glycosphingolipids and sphingomyelin, are essential for synapse development, stabilization, and maintenance [12], [18]. Moreover, caveolin-1 (Cav-1), a cholesterol binding and resident protein of MLR [19], [20], [21], organizes and targets synaptic components of the neurotransmitter and neurotrophic receptor signaling pathways to MLR [e.g., NMDAR, AMPAR, TrkR, Src Family Kinases (SFK)] [22], [23], [24], [25], [26], [27]. Additionally, neurotransmitter and neurotrophic receptors are found within MLR in growth cones, a finding that has major implications for neuronal plasticity [11], [28].

Early-onset AD, which afflicts individuals prior to 60–65 years of age, is known to be caused by mutations in three genes: amyloid precursor protein (APP), presenilin-1, and presenilin-2 [29]. MLR and cholesterol play a protective role against APP processing and amyloid-β (Aβ) toxicity [13], [14], [16], [30], [31], [32], [33]. Cav-1 KO mice develop CNS pathology similar to AD, such as altered NMDA receptor signaling, motor and behavioral abnormalities, increased ischemic cerebral injury, impaired spatial memory, and cholinergic function [27], [34], [35], [36]. Whether MLR, Cav-1 expression, and the organization of pro-survival and pro-growth signaling mechanisms are altered in neurodegenerative states (age-related dementia and AD) has yet to be investigated. The present study tested whether 1) Cav-1 organizes synaptic signaling components in neuronal MLR and synaptosomes, 2) the localization of synaptic signaling components to neuronal MLR and synaptosomes is reduced in brains from aged wild-type and young Cav-1 KO mice, and 3) brains from Cav-1 KO mice develop a neuropathological phenotype similar to Alzheimer's disease.

## Results

### PSD-95, NR2A, NR2B, and Cav-1 protein expression is decreased in middle aged and aged hippocampus

**Figure 1:**
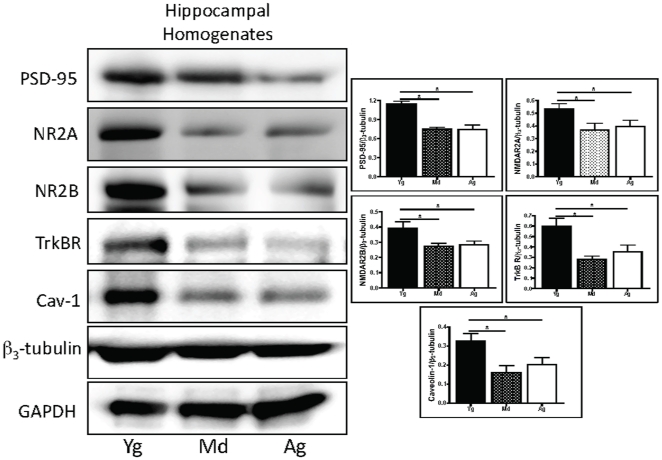
Hippocampal homogenates show an aged dependent reduction in NR2A, NR2B, PSD-95, and Cav-1. Hippocampi were isolated from the brains of C57BL/6J mice at 3–6 months (young, Yg), 12 months (middle aged, Md), and 24 months (aged, Ag). Immunoblot and densitometric analysis demonstrated a significant reduction in PSD-95, NR2A, NR2B, TrkBR, and Cav-1 in the Md and Ag hippocampus compared to Yg.

Hippocampi were isolated from brains of C57BL/6J mice (wild-type, WT) at 3–6 months (young), 12 months (middle aged), and >18 months (aged). Immunoblots of hippocampal homogenates showed a significant reduction in PSD-95 (n = 6, p = 0.0001 vs Md, p = 0.01 vs Ag), NR2A (n = 6, p = 0.02 vs Md, p = 0.02 vs Ag), NR2B (n = 6, p = 0.02 vs Md, p = 0.04 vs Ag), TrkB (n = 6, p = 0.009 vs Md, p = 0.03 vs Ag), and Cav-1 (n = 6, p = 0.008 vs Md, p = 0.04 vs Ag) in hippocampi from middle aged and aged mice when compared to young mice (Figure 1). These data demonstrate an age-dependent reduction in synaptic signaling components and Cav-1 in the hippocampus.

### Age-related decreases in synaptic signaling components from MLR

**Figure 2:**
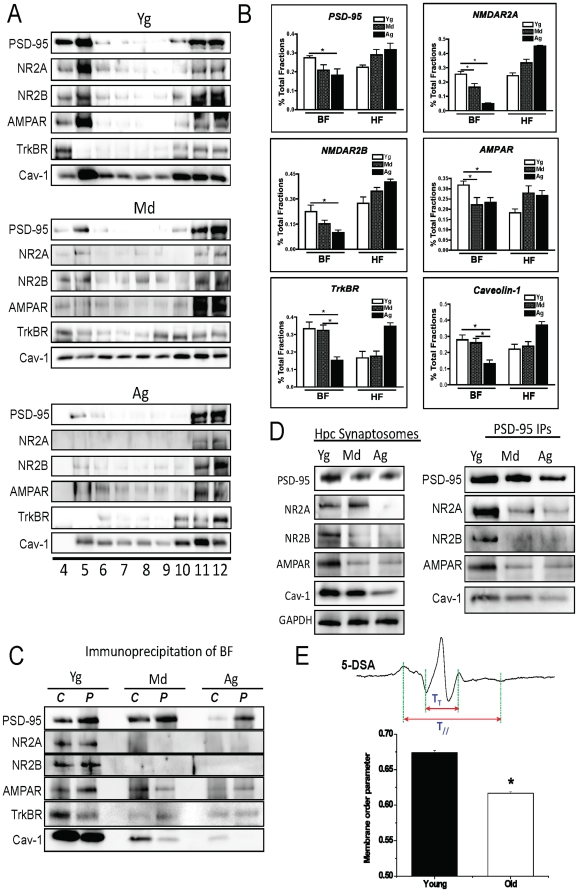
PSD-95, NR2A, NR2B, AMPAR, TrkBR, and Cav-1 are abundantly detected in buoyant fractions (BF) from young mouse brains homogenates, yet are less abundant BFs from middle aged and aged brains. Sucrose density fractionated was performed on brains from three different age groups of C57BL/6J mice: young (Yg, 3–6 months), middle aged (Md, 12 months), and aged (Ag, >18 months). Immunoblot analysis detected the majority of PSD-95 (post-synaptic density marker), NR2A, NR2B, AMPAR, TrkBR, and Cav-1 in buoyant fractions 4 and 5 (BFs) isolated from Yg brains (**A**). In contrast, the Md and Ag brains exhibited a drastic reduction in these synaptic signaling components, with the majority of these proteins detected in heavy fractions 11 and 12 (HFs) only. Densitometric analysis of the data is represented in **B**. (**C**) Cav-1 (***C***) and PSD-95 (***P***) immunoprecipitates pulled down NR2A, NR2B, AMPAR, and TrkB in the buoyant fractions of Yg mice, with decreased detection in Md and Ag. (**D**) Immunoblot analysis detected a significant decrease in PSD-95 (post-synaptic density marker), NR2A, NR2B, AMPAR, and Cav-1 in hippocampal synaptosomes from Md and Ag brains compared to Yg. PSD-95, NR2, NR2B, AMPAR, and Cav-1 decreased in PSD-95 immunoprecipitates of Md, and Ag synaptosomes compared to Yg. (**E**) Electron paramagnetic resonance (EPR) was performed on synaptosomal membranes from brains of C57BL/6J mice: young (Yg, 3–6 months) and aged (Old, >18 months). Membrane localized spin labels 5-doxylstearic acid (5-DSA) probes changes in the neuronal membrane fluidity closer to the membrane surface. Lineshape analysis of 5-DSA spin label using the indicated parameters revealed that neuronal membrane of aged mice exhibit significantly lower order parameter (i.e. increased fluidity) than young animals. Aged membranes were 8.5±1.2% more fluid than young membranes (F_(1,10)_ = 223.5, p = 0).

MLR play a role in stabilizing synapses in the mammalian brain [12], [18], therefore we performed sucrose density fractionation of whole brain homogenates from young, middle aged and aged WT mice to purify MLR. Immunoblots showed buoyant fractions from young brains contained the majority of PSD-95 (n = 5, p = 0.03 vs Ag), NR2A (n = 5, p = 0.04 vs Md, 0 = 0.0005 vs Ag), NR2B (n = 5, p = 0.01 vs Ag), AMPAR (n = 5, p = 0.04 vs Md, 0 = 0.02 vs Ag), TrkB (n = 5, p = 0.005 vs Ag, 0 = 0.003 Md vs Ag), and Cav-1 (n = 5, p = 0.004 vs Ag, 0 = 0.004 Md vs Ag) (Figure 2A,B). In contrast, buoyant fractions from the middle aged and aged brains showed a significant reduction in synaptic signaling components compared to Yg, with the majority of the proteins detected in heavy fractions, 11 and 12 only. Cav-1 (***C***) and PSD-95 (***P***) co-immunoprecipitated with NR2A, NR2B, AMPAR, and TrkB in the buoyant fractions of Yg mice, with decreased detection in Md and Ag (Figure 2C). These data demonstrate an age-dependent decrease in synaptic signaling components including Cav-1 from MLR and PSD-95 immunoprecipitation of MLR.

Previous work has shown that MLR facilitate neuronal synapse formation [12], [18]. We sought to confirm whether the age-related decrease in synaptic signaling components in MLR also occurred in synaptosomes purified from hippocampi of Yg, Md, and Ag WT mice. Immunoblots and PSD-95 immunoprecipitates of synaptosomes from Md and Ag mice showed a decrease in PSD-95, NR2A, NR2B, AMPAR, and Cav-1 compared to Yg (Figure 2D). Assessment of membrane fluidity of synaptosomes isolated from whole brain of Yg and Ag mice using electron paramagnetic resonance showed that membranes of Ag mice had significantly lower membrane order parameter (greater fluidity) than membranes from Yg (n = 5, p = 0.001) (Figure 2E). These findings suggest that age-related decrease in MLR and Cav-1 expression are associated with increased membrane fluidity (i.e., increased liquid-disordered phase) [37].

### Young Cav-1 KO mice demonstrate accelerated aging and neurodegeneration

#### Loss of synaptic proteins and neuronal preconditioning

**Figure 3:**
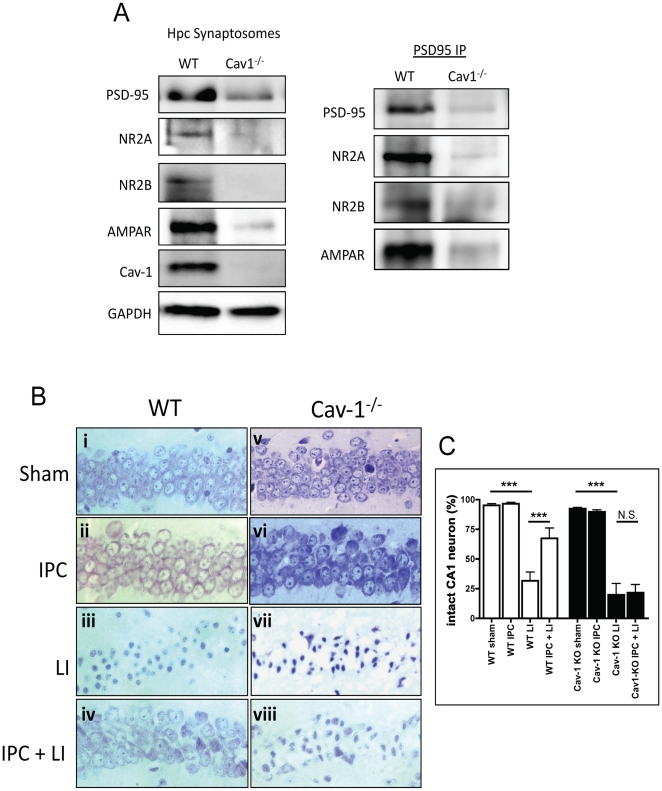
Ischemic preconditioning (IPC) does not occur in Cav-1 KO mice. (**A**) Hippocampal synaptosomes from Cav-1 KO (Yg) showed a similar pattern to Ag, with a decrease in PSD-95, NR2A, NR2B, and AMPAR. PSD-95 IPs of Cav-1 KO synaptosomes revealed minimal detection in PSD-95, NR2A, NR2B, and AMPAR. (**B**) WT or Cav-1 KO mice were subjected to 3 min (ischemic preconditioning, IPC) and/or 12 min (lethal ischemia, LI) induced by bilateral carotid artery occlusion (BCAO). Intact neurons in CA1 hippocampal (HP) region were counted from Cresyl Violet stained paraffin fixed sections. IPC (3 min, BCAO) significantly protected CA1 neurons against LI (12 min, BCAO) in WT mice (**iv**). There was a significant increase in CA1 neuronal death in Cav-1 KO animals subject to IPC (**viii**) versus WT IPC + LI. Representative Cresyl Violet stained CA1 hippocampal images from (**i**) WT sham, (**ii**) WT IPC, (**iii**) WT LI, and (**iv**) WT IPC and (**v**) Cav-1 KO sham, (**vi**) Cav-1 KO IPC, (**vii**) Cav-1 KO LI, and (**viii**) Cav-1 KO IPC. Quantitation of images is presented by the graph.

Cav-1 expression is decreased in hippocampi and buoyant fractions (i.e., MLR) from Ag mice (Figure 1 and 2), we therefore assessed whether Yg Cav-1 KO mice display reduced synaptic protein expression. Hippocampal synaptosomes from Yg Cav-1 KO mice showed a similar pattern to Ag WT mice, reduced protein expression of PSD-95, NR2A, NR2B, and AMPAR (Figure 3A). Similar to aged WT mice, PSD-95 immunoprecipitation of hippocampal synaptosomes from Cav-1 KO mice showed minimal detection of PSD-95, NR2A, NR2B, and AMPAR.

We next sought to determine whether neuroprotection against ischemic injury is absent in Yg Cav-1 KO mice. To achieve this we performed an ischemic preconditioning protocol. Ischemic preconditioning (IPC), a phenomenon wherein sublethal ischemia protects the brain from a subsequent lethal ischemic event, is absent in brains from aged animals [38], [39] and in neurons *in vitro* that have reduced or no Cav-1 expression [27]. We show here for the first time that Cav-1 KO mice show a similar reduction in neuroprotective signaling components to that exhibited by brains from aged WT mice. IPC significantly protected CA1 neurons against lethal ischemia in WT mice (n = 7, p = 0.0072 vs LI) (Figure 3B -iv, C). There was no significant protection in CA1 neurons from Cav-1 KO mice subjected to IPC prior to LI (Figure 3B -viii, C), demonstrating an inability to induce IPC in these mice. In terms of expression and function of synaptic signaling components, young Cav-1 KO mice resemble aged WT mice.

### Early on-set of AD-like phenotype

**Figure 4:**
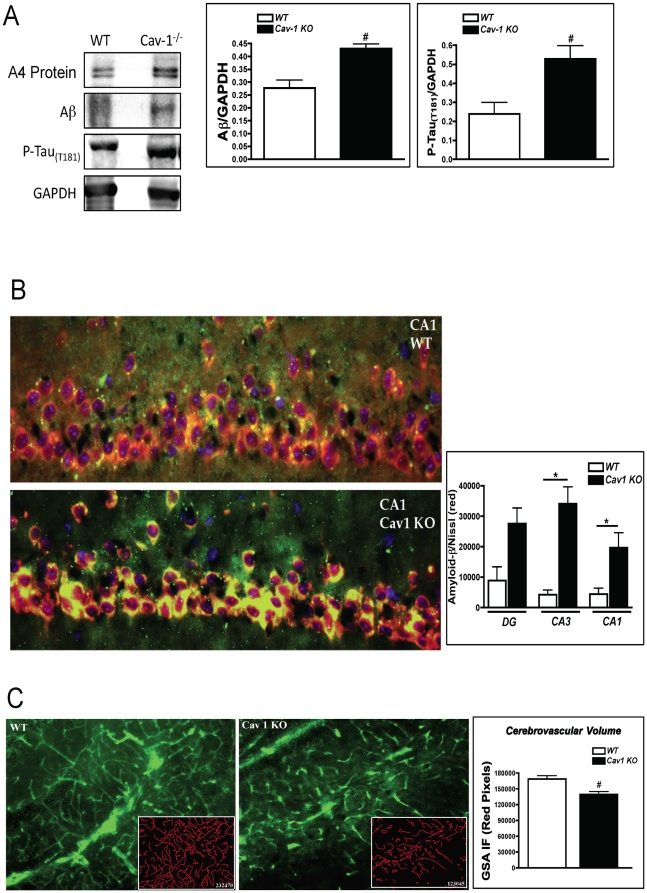
Aβ, A4 protein, and P-Tau(T181) are elevated in the hippocampus of young Cav-1 KO mice. (**A**) Hippocampal homogenates from WT (3 m) and Cav-1 KO (Cav-1 KO, 3–6 m) C57Bl/6J mice were immunoblotted for Aβ and phosphorylated Tau (P-Tau_[T181]_), and GAPDH. Aβ and P-Tau_[T181]_ were significantly elevated in young Cav-1 KO hippocampal homogenates. (**B**) Immunofluorescence microscopy showed that Cav-1 KO CA1 region of the hippocampus displayed elevated Aβ staining (green) overlapping with Nissl positive neurons (red) as indicated by yellow fluorescence. Quantitation of the data is represented in the graph. (**C**) Cryostat sections (50 µm) of mouse hippocampus were stained with lectin GSA (Griffonia simplicafolia) to label blood vessels. There was a 20–25% reduction in overall area occupied by blood vessels in Cav-1 KO. Quantitation of the data is represented in the graph (*right*).

Previous work has shown that Cav-1 and MLR can regulate amyloidogenic processing of APP [30]. Therefore, we assessed whether brains from Cav-1 KO mice exhibit pathological signs indicative AD. Amyloid-β (Aβ) (n = 4, p = 0.005) and P-Tau_[T181]_ (n = 4, p = 0.02) were significantly elevated in hippocampal homogenates from Yg Cav-1 KO mice (Figure 4A). Immunofluorescence microscopy demonstrated that Yg Cav-1 KO mice had increased Ab staining in Nissl positive neurons in the CA3 (n = 3, p = 0.006) and CA1 (n = 3, p = 0.04) region of the hippocampus compared to WT mice (Figure 4B). Hippocampi from Cav-1 KO mice showed a 20–25% reduction in cerebrovascular volume (n = 4, p = 0.001) (GSA, blood vessel marker - Figure 4C).

**Figure 5:**
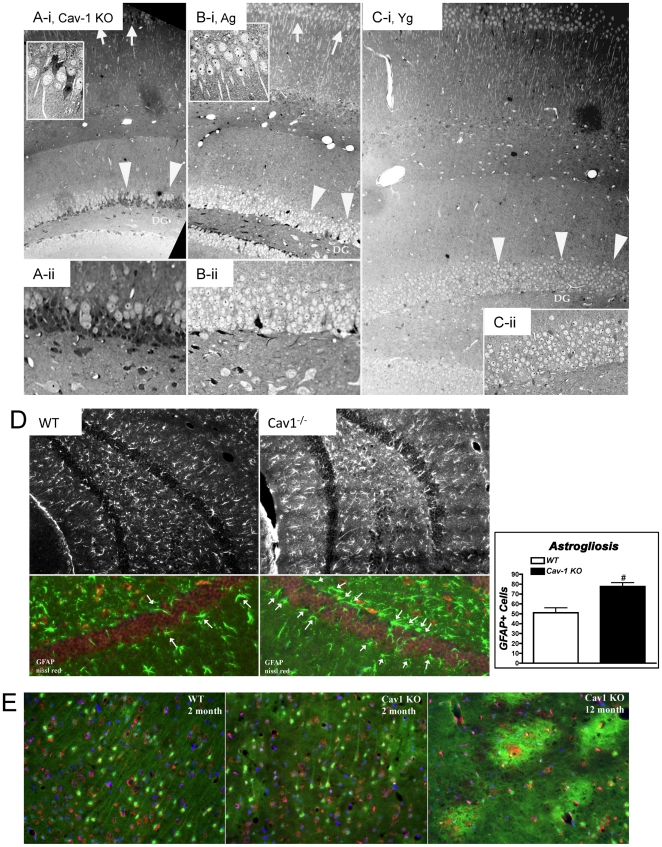
Cav-1 KO mice exhibit enhanced astrogliosis and neuronal degeneration. (**A–C**) Light microscopic image displaying 0.5 µm thick hippocampal sections of Cav-1 KO (**A** *-i,* **A** *-ii*), aged (**B** *-i,* **B** *-ii*), and young (**C** *-i,* **C** *-ii*) stained with toludine blue. There is a drastic reduction in neurons within the dentate gyrus (large arrow heads) and CA1 regions (arrows) of young Cav-1 KO mice compared to young and aged WT. In addition, there appears to be the presence of more glia and glial scar formation within the dentate gyrus of Cav-1 KO mice as indicated by the darker gray cell bodies intermixed with the neurons. (**D**) Hippocampal coronal cryostat sections (10 µm) from WT and Cav-1 KO mice were stained with Nissl (neuronal marker, red pixels) and GFAP (astrocyte marker, green) to show no overlap between neurons and astrocytes. (**E**) Coronal cryostat sections (25 µm) of 2 month WT, 2 month Cav-1 KO and 12 month Cav-1 KO stained with 0.0004% *Flouro-Jade®B* and fluorescent red Nissl with DAPI. Areas from CA1 of the hippocampus were imaged. WT CA1 showed well-organized astrocytes. Two month Cav-1 KO had areas of disorganized astrocytes with lightly labeling areas of potential future plaque development. Twelve month Cav-1 KO CA1 areas had large bright, entangled green fluorescence with red neurons inside and significantly less organized astrocytes, further demonstrating a degenerating neuronal model.

Toludine blue staining of the hippocampus showed a large reduction in neurons within the dentate gyrus and CA1 regions of Yg Cav-1 KO mice (Figure 5A -i, A-ii) compared to Yg (Figure 5C -i, C-ii) and Ag (Figure 5B -i, B-ii) WT mice. In addition, there appeared to be more glia and glial scar formation within the dentate gyrus of Cav-1 KO mice as indicated by the darker gray cell bodies intermixed with the neurons (Figure 5A -i, A-ii). Young Cav-1 KO show increased astrogliosis (n = 4, p = 0.0006) (GFAP, astrocyte marker – Figure 5D). *Flouro-Jade®B* staining demonstrated little neuronal degeneration and well-organized astrocytes in the CA1 from Yg WT mice when compared with Yg Cav-1 KO mice, which showed disorganized astrocytes and areas of potential plaque development. Due to their shorter life spine [40], obtaining Ag Cav-1 KO mice is difficult. We here show that the CA1 region from 12 month Cav-1 KO mice had large bright, entangled green fluorescence with red fluorescent (Nissl) neurons and severely less organized astrocytes, demonstrating increased neuronal degeneration (Figure 5E).

**Figure 6:**
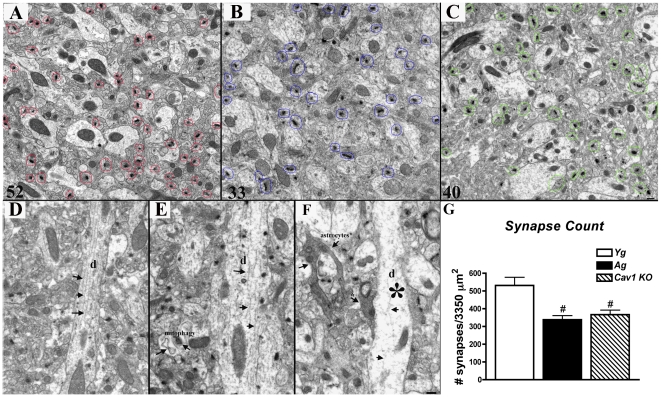
Cav-1 KO mice have reduced hippocampal synapses. Synapses were quantified by routine electron microscopy as previously described [82]. EM analysis revealed a significant reduction in hippocampal synapses in both (**C**) Cav-1 KO (Yg) and (**B**) Ag mice compared to (**A**) WT. Synapses are indicated by red circles in WT, blue circles in Ag, and green circles in Cav-1 KO. (**D**) WT micrographs exhibited dendritic processes (indicated by d) with intact cytoskeletal architecture (arrows and arrowheads), while (**E**) Ag and (**F**) Cav-1 KO displayed less organized dendritic shafts (asterisk) with more abundant astrocyte presence (arrows). (**G**) Quantitation of data.

There is a reduction in synaptic proteins from hippocampal synaptosomal membranes, we therefore assessed whether Cav-1 KO mice exhibit changes in total hippocampal synapses. Routine electron microscopy (EM) revealed a significant reduction in hippocampal synapses (i.e., post synaptic densities) in both Cav-1 KO (n = 6, p = 0.002) (Figure 6C) and Ag (n = 6, p = 0.02) (Figure 6B) mice compared to Yg (Figure 6A). In addition, Cav-1 KO mice displayed unorganized cytoskeletal assemblage (**arrow heads**) within dendrites (**d, asterisks**) (Figure 6F) and elevated astocyte presence (**arrows**) compared to brains from Ag (Figure 6E) and Yg WT mice (Figure 6D), the latter displaying normal cytoskeletal organization (**arrow heads**) within dendrites (**d**). These data indicate that Cav-1 KO mice develop pathological changes at 3 months of age consistent with aging and AD mouse models.

### Re-expression of Cav-1 in Cav-1 KO neurons decreases Aβ

**Figure 7:**
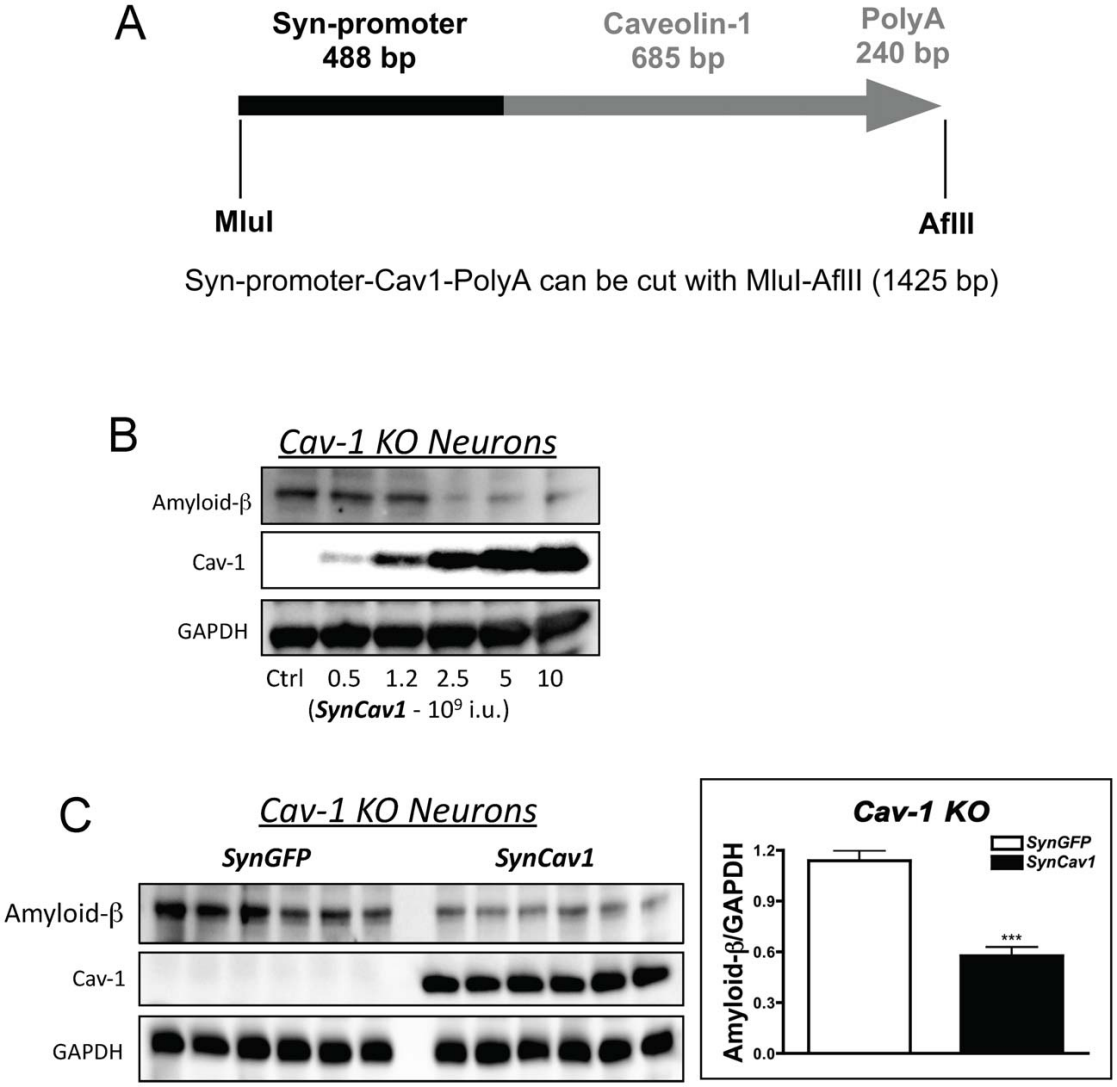
Neuron-targeted re-expression of Cav-1 reduces Aβ expression in primary neurons cultured from Cav-1 KO brains. Primary neurons from Cav-1 KO mice were grown in culture for 4 days and transfected a lentiviral vector containing Cav-1 driven by the synapsin promoter (*SynCav1*) for 72 hr. *SynGFP* served as control vector (*2×*10^9^ viral particles for both vectors). Schematic of the vector is shown in **A**. Increasing doses of *SynCav1* proportionally decreased Aβ expression (**B**). Six separate primary cultures of Cav-1 KO neurons were incubated with either *SynGFP* or *SynCav1*. *SynCav1* significantly decreased Aβ expression after 72 hr.

Cav-1 KO mice demonstrate pathology similar to AD such as elevated Aβ production in the hippocampus. We tested whether neuron-targeted re-expression of Cav-1 in primary Cav-1 KO neurons would decrease Aβ expression. We generated a viral vector that contains a neuron-specific synapsin promoter upstream of Cav-1 cDNA (*SynCav1*) (Figure 7A). Increasing doses of *SynCav1* for 72 hr proportionally increased Cav-1 expression and reduced Aβ (Figure 7B). Six separate neuronal cultures from Cav-1 KO mouse brains were transfected with *SynGFP* (control vector) or *SynCav1*, and *SynCav1* significantly reduced Aβ expression (n = 6, p = 0.002) after 72 hr (Figure 7C).

## Discussion

The present study is the first to demonstrate that the cholesterol binding and MLR resident protein, Cav-1, complexes with synaptic proteins in the CNS, and that this organization is disrupted with age. Furthermore, this study is the first to demonstrate that loss of Cav-1 in a transgenic mouse model produces neuropathology similar to that exhibited with AD, i.e., Aβ production, elevated astrogliosis, reduced cerebrovasculature and neuronal loss in the hippocampus. Our data suggest that not only are MLR and Cav-1 essential for maintaining and stabilizing proper synaptic signaling [27] and neuroprotection against cerebral ischemia, but they also may serve to slow the amyloidogenic process of APP seen in AD brains. Lastly, Cav-1 KO mice may serve as the first non-mutational model of AD.

It is essential to understand the basic neural mechanisms of synapse formation and stabilization in order to identify potential therapeutic targets for facilitating neuronal regeneration and recovery of neuronal networks in the aged and injured brain. Traditionally synapses and MLR are considered separate subcellular structures, yet they both contain identical physical characteristics that are essential such as cholesterol, glycosphingolipids, sphingomyelin, and other saturated fatty acid containing lipids (GM_1_ gangliosides, palmitic acid) as well as signaling components [22], [23], [24], [25], [26], [27]. Growing evidence supports the role for free cholesterol and MLR in neuronal synaptic formation, signaling and protection [12], [18], [27], [41], [42], [43]. Because free cholesterol directly affects Cav-1 expression, factors that alter intracellular cholesterol also change Cav-1 expression [44], [45], [46]. Specifically, brain derived neurotrophic factor (BDNF), a neurotrophin essential to synaptic function and development [47] which facilitates of long-term potentiation[48], [49], elicits cholesterol biosynthesis and increased MLR and Cav expression in cortical and hippocampal neurons.[50] Furthermore, MLR are critical for growth cone expansion, neurite outgrowth, and axonal branching and guidance [11], [51], [52]. Therapeutic approaches to promote axonal regeneration and synapse formation after spinal chord injury use a MLR marker, cholera toxin B, as a direct indicator of axonal regeneration and de novo synapse formation [53], [54]. Moreover, there exists increasing evidence that disruption or alterations of neuronal MLR and intracellular cholesterol can be neurotoxic and even contribute to enhanced neuronal vulnerability to Ab [13], [14], [33], demonstrating the importance of these distinct microdomains for proper pro-survival neuronal signaling [27], [41], [55], [56], [57]. When Cav-1 was over-expressed in β-secretase expressing cells, amyloid precursor protein and β-secretase localization to MLR resulted in decreased Aβ production, suggesting a protective role by Cav-1 and MLR against Aβ toxicity [30], [31], [32], [58]. Interestingly the fatty acid content in MLR (a.k.a. detergent-resistant membranes, or DRMs) isolated from synaptic endings is altered in aged animals [59]. This result is consistent with our findings that membrane fluidity in synaptosomal membranes is increased in aged brains. Age-related physiochemical changes to distinct biological membranes such as MLR could be responsible for changes in Cav-1 expression and loss of synaptosomal pro-survival signaling components with age.

Our results demonstrate that loss of Cav-1 results in accelerated aging. Cav-1 KO mice have a shortened life span [40]. Two pathophysiologies altered with aging are vulnerability to ischemic stress and progression of AD. IPC is a phenomenon whereby brief ischemia, which does not injure neurons, renders the brain less vulnerable to subsequent ischemic injury [27], [60], [61], [62], [63], [64]. IPC activates endogenous signaling pathways that are neuroprotective, and this neuroprotection is lost in the aged brain [38], [39]. The underlying mechanism for the lack of ischemic tolerance in the aged brain is not clear. Signaling pathways in neurons are severely compromised with age. Specifically, post-synaptic molecules such as glutamate receptors, neurotrophin receptors and pro-survival signaling cascades (i.e., kinase activation and cAMP production) decrease significantly with age [65], [66], [67], [68], [69]. It is therefore possible that the organization, and thus efficacy of signaling pathways that produce tolerance is severely limited in the aged brain. We show in young Cav-1 KO mice that preconditioning is absent, suggesting a link between the loss of MLR and disrupted organization of pro-survival signaling.

In addition to loss of IPC, Cav-1 KO mice also exhibit characteristics consistent with AD. Cerebrovascular changes and increased astrogliosis [70], [71], [72], [73], [74] could also be a contributing factor to the absence of ischemic tolerance [75] as well as the AD phenotype exhibited by young Cav-1 KO mice. Upregulation of endogenous protective signaling in aged neurons through neuron-targeted Cav-1 expression might reduce the vulnerability of the aged brain even in the presence of reduced cerebrovascular volume. Neuron-targeted Cav-1 re-expression/over-expression offers the novel possibility of re-establishing the fidelity of neuroprotective signaling that is lost with advanced age or in other forms of neurodegeneration (i.e., dementia, Alzheimer's disease, depression, Parkinson's disease).

In summary, these findings demonstrate an important role for Cav-1 and MLR in organizing synaptic pro-survival signaling components that are essential for neuroprotection against ischemic injury, neuronal regeneration, and maintaining synapse stabilization and formation. Cav-1 may be a control point for neurological aging. Further understanding of how MLR and Cav-1 serve as a nexus for pro-survival and pro-growth signaling components may not only provide potential therapeutic targets for the preservation of neuronal function, but may also yield tools that could augment the brain's capacity to reorganize its neuronal networks following injury or during late stages of neurodegenerative diseases such as AD and other forms of dementia.

## Materials and Methods

All studies performed on animals were approved by Veteran Affairs San Diego Institutional Animal Care and Use Committee (Protocol#: 08-035 and ID#:1141788) and conform to relevant National Institutes of Health guidelines.

### Primary neuron isolation and culture

Neonatal mouse neurons were isolated using a papain dissociation kit (Worthington Biochemical, Lakewood, NJ) as previously described [27]. Neurons were cultured in Neuobasal A media supplemented with B27 (2%), 250 mM GLUTMax1, P/S (1%). Cells were cultured on poly-D-lysine/laminin (2 µg/cm^2^) coated plates at 37°C in 5% CO_2_ for 4 d prior to transfection with lentiviral vectors. Cav-1 cDNA was cloned in our laboratory and given to Dr. Atushi Miyanohara at the UCSD Viral Vector Core. Dr. Miyanohara successfully generated a lentiviral vector containing the synapsin promoter up-stream of the *Cav-1* gene (*SynCav1*). *SynGFP* was used as control vector. Titer for both vectors was approximately 10^9^ infectious units (i.u.) per ml.

### Sucrose-density fractionation

Membrane/lipid rafts were isolated from adult brain and neurons using detergent-free methods. Tissue and cells were homogenized in sodium carbonate (150 mM, pH 11.0), and then sonicated with three cycles of 20 sec bursts with 1 min incubation on ice. Homogenate (1 mL) was mixed with 1 mL of 80% sucrose to generate 2 mL of 40% sucrose. Above the 40% layer, 6 mL of 35% and 4 mL of 5% sucrose were carefully layered. The mixture was centrifuged at 175,000 g using SW41Ti rotor (Beckman) for 3 h at 4°C. Samples were removed in 1 ml aliquots and the membrane/lipid rafts are found in buoyant fractions 4–5 (5/35% interface).

### Synaptosomal membrane preparation

Neuronal cells or brain tissue were homogenized in 5 ml of solution A [0.32 M sucrose (34 g/500 ml), 0.5 mM CaCl_2_ (36 mg/500 ml), 1 mM NaHCO_3_ (42 mg/500 ml), 1 mM MgCl_2_ (102 mg/500 ml)] containing protease and phosphatase inhibitors with 12 strokes of a 19×84 mm tissue grinder (Potter Elvehjem, plastic coated) at 800 r.p.m. Samples were then subjected to centrifugation for 10 min at 1000 g at RT to remove large debris. **Centrifugation 1** involved careful layering of the supernatant onto 4 ml of 1.2 M sucrose (41 g/100 ml or 41% sucrose) in a SW41 centrifuge tube (Beckman) and then spun at 160,000 g for 15 min (or 33,000 r.p.m. with SW41 rotor). The synaptosomes were found at the interface between the 1.2 M and 0.32 M sucrose layers. The synaptosomes were then mixed with 4 ml of 0.32 M sucrose and then carefully layered onto 4 ml of 0.8 M sucrose (or 27% sucrose) in a fresh centrifuge tube for second major centrifugation. **Centrifugation 2** consisted of spinning the sample at 160,000 g for 15 min (33,000 rpm with SW41 rotor) generating a pellet enriched in the synaptosomes. The pellet was then resuspended in 1 ml of neuronal lysis buffer containing protease and phosphatase inhibitors and used for immunoprecipitation and/or immunoblot analysis.

### Determination of synaptosomal membrane fluidity using electron paramagnetic resonance (EPR)

Hydrocarbon chain mobility was measured using fatty acid spin labeling EPR analysis using 5-nitroxyl stearate (5-DSA, Aldrich) as a spin probe [76], [77]. The number designation indicates the relative position of the nitroxide on the stearic acid relative to the polar carboxylic group. In the case of 5-DSA, the spin probe is firmly held in place by the head groups of the lipids, which is reflected in broad EPR lines. Synaptosomes from young (3–6 m) and aged (>18 m) mice were isolated as described previously [78]. Freshly prepared synaptosomal protein (0.1–0.2 mg) was incubated for 15 minutes with 5-DSA (1 mM final concentration) in synaptosomal buffer (120 mM NaCl, 4.7 mM KCl, 2.2 mM CaCl_2_, 1.2 mM MgCl_2_, 25 mM HEPES, 1.2 mM MgSO_4_, 1.2 mM KH_2_PO_4_, 10 mM glucose) at 25°C. The mixture was then loaded into a 50 µl-glass capillary and inserted into the EPR cavity of a MiniScope MS200 Benchtop spectrometer (Magnettech, Berlin), maintained at 37°C, where the EPR spectra registered. EPR conditions were the following: microwave power, 5 mW; modulation amplitude, 2 G; modulation frequency, 100 kHz; sweep width, 150 G centered at 3349.0 G; scan rate, 7.5 G/s, with each spectrum representing the average of 5 scans. The fluidity parameters *T*_||_ and *T*_⊥_ are defined in **Figure 2E** and are used to calculate the order parameter as previously described [76], [77].

### *In vivo* BCAO (bilateral carotid artery occlusion) model of neuronal preconditioning

Male C57BL/6J and Cav-1 KO mice were anesthetized with isoflurane. After endotracheal intubation, the lungs were mechanically ventilated with 1.5% isoflurane in 30% O_2_, balanced N_2_. Pericranial temperature was controlled at 37°C. Via a pre-tracheal incision, the carotid arteries and the basilar artery were exposed and a temporary clip was applied to the basilar artery. Thereafter, preconditioning (PC) was induced by occlusion of the carotid arteries. The clips were removed after a defined interval (3 min for PC and 10 min for lethal ischemia), the wounds were infiltrated with 0.25% bupivacaine and the anesthetic was discontinued. Upon resumption of spontaneous ventilation, the endotracheal tube was removed and the animals were transferred to the animal care facility 4 hr post extubation. Animals underwent transcardiac perfusion with heparinized saline followed by buffered paraformaldehyde. The brains were removed and the extent of injury to the CA1 sector of the hippocampus was determined by Cresyl violet staining.

### Routine and immunoelectron microscopy

Brains were transcardially perfusion fixed with standard Karnovsky's fix, 4% paraformaldehyde, 1% gluteraldehyde, 0.1 M cacodylate buffer with 5 mM CaCl_2_. PND5-7 animals were fixed with 2% paraformaldehyde, 2.5% glutaraldehyde, 0.1 M cacodylate buffer and 5 mM CaCl_2_ to prevent tissue artifacts. Hippocampi were dissected from whole brains after 24 h and 400 µm vibratome slices prepared and re-fixed an additional 24 h. Brains were blocked (i.e., dissected) to include hippocampal areas, one hemisphere for sagittal orientation, and one hemisphere for coronal. Blocks were re-fixed for an additional 24 h followed by post-fixation with 1% OsO_4_ in 0.1 M cacodylate buffer, *en bloc* stained with uranyl acetate and embedded with flat orientation to locate appropriate hippocampal regions of interest. Each block was thick sectioned, stained with toludine blue, and re-trimmed to isolate hippocampal areas prior to preparation of grids. Grids (70 nm sections) were stained with uranyl acetate and lead nitrate for contrast and observed on the electron microscope [JEOL 1200 EX-II (Tokyo, Japan)] equipped with a digital camera system. 25 random low magnification micrographs of the stratum radiatum were obtained from each specimen. Micrographs were analyzed for the quantity of synapses and for synapse abnormalities (reduction or changes in synapse and dendritic filopidal spine morphology, i.e., degradation of cytoskeletal architecture). The dendritic profiles were characterized by abundant organelles such as mitochondria and endoplasmic reticulum and frequent contacts from vesicle-filled axon terminals. Spine synapses were identified by an electron dense region associated with vesicles pre-synaptically and that lacked cellular organelles or contained a spine apparatus (as indicated by cytoskeletal architecture) with post-synaptic densities as described previously [79], [80], [81], [82]. Approximately 25 electron micrographs (3350 µm^2^) per animal were analyzed in a blinded fashion for total synapse number per area (synapse #/3350 µm^2^).

### Generation of *SynCav1* construct

To link the neuron-specific synapsin (Syn) promoter with the Cav1 cDNA, XbaI-SalI DNA fragment containing the Syn promoter was inserted into the NheI-SalI sites of the pEGFP-N1 (Clontech) and the resulting plasmid was designated pSyn-EGFP. A 685bp Cav1 cDNA was isolated from the pCRII-TOPO vector (Invitrogen) by PmeI-NotI digest and inserted into the SmaI-NotI site of the pSyn-EGFP to generate the pSyn-Cav1, in which the EGFP gene was replaced with the Cav1 cDNA. The Syn-promoter-Cav1 cassette was isolated from the pSyn-Cav1 and inserted into the BamHI site of the HIV1 vector backbone plasmid pHIV7 [83] and the resulting plasmid was designated pHIV1-Syn-Cav1.

### Statistics Analysis

All parametric data were analyzed by unpaired t-tests or ANOVA Bonferroni's Multiple Comparison as appropriate; post hoc comparisons were made by Student Neuman Keuls tests. Significance was set at p<0.05. Statistical analysis was performed using Prism 4 (GraphPad Software, Inc., La Jolla, CA).
